# Epigenome Mapping Identifies Tumor-Specific Gene Expression in Primary Rectal Cancer

**DOI:** 10.3390/cancers11081142

**Published:** 2019-08-09

**Authors:** Hannah Flebbe, Feda H. Hamdan, Vijayalakshmi Kari, Julia Kitz, Jochen Gaedcke, B. Michael Ghadimi, Steven A. Johnsen, Marian Grade

**Affiliations:** 1 Department of General, Visceral and Pediatric Surgery, University Medical Center Goettingen, 37075 Goettingen, Germany; 2 Gene Regulatory Mechanisms and Molecular Epigenetics Laboratory, Division of Gastroenterology and Hepatology, Mayo Clinic, Rochester, MN 55905, USA; 3 Institute of Pathology, University Medical Center Goettingen, 37075 Goettingen, Germany

**Keywords:** ChIP-seq, chromatin, epigenetics, histone modification, H3K27ac, rectal cancer, gene expression

## Abstract

Epigenetic alterations play a central role in cancer development and progression. The acetylation of histone 3 at lysine 27 (H3K27ac) specifically marks active genes. While chromatin immunoprecipitation (ChIP) followed by next-generation sequencing (ChIP-seq) analyses are commonly performed in cell lines, only limited data are available from primary tumors. We therefore examined whether cancer-specific alterations in H3K27ac occupancy can be identified in primary rectal cancer. Tissue samples from primary rectal cancer and matched mucosa were obtained. ChIP-seq for H3K27ac was performed and differentially occupied regions were identified. The expression of selected genes displaying differential occupancy between tumor and mucosa were examined in gene expression data from an independent patient cohort. Differential expression of four proteins was further examined by immunohistochemistry. ChIP-seq for H3K27ac in primary rectal cancer and matched mucosa was successfully performed and revealed differential binding on 44 regions. This led to the identification of genes with increased H3K27ac, i.e., *RIPK2*, *FOXQ1*, *KRT23*, and *EPHX4*, which were also highly upregulated in primary rectal cancer in an independent dataset. The increased expression of these four proteins was confirmed by immunohistochemistry. This study demonstrates the feasibility of ChIP-seq-based epigenome mapping of primary rectal cancer and confirms the value of H3K27ac occupancy to predict gene expression differences.

## 1. Introduction

Colorectal cancer (CRC) represents the third most common cancer type and the second leading cause of cancer-related death in the western world [1]. The stepwise progression from normal epithelium to premalignant lesions and, ultimately, to invasive adenocarcinomas is characterized by the accumulation of specific genetic alterations, chromosomal aneuploidies and accompanying gene expression changes [2,3,4,5]. Over the last two decades, CRC has been extensively characterized with respect to mRNA and microRNA expression profiles, DNA sequence and copy number changes, and proteomic signatures [6,7]. Such analyses have led to the identification of consensus molecular subtypes of CRC [8,9]. More recently, other studies have focused on understanding the underlying epigenetic changes associated with CRC, with a particular focus on aberrant DNA methylation [10,11].

Alterations in chromatin, including changes in histone modifications or their modifying enzymes or changes in the expression or activity of chromatin remodelers due to genetic alterations, also frequently occurs in cancer, but are more poorly understood [11,12]. To date, most studies examining changes in histone modifications have largely been performed in cultured cells, precluding a clear interpretation of the clinical importance of these analyses. We therefore aimed to assess the potential utility of chromatin immunoprecipitation (ChIP) followed by next-generation-sequencing (ChIP-seq) for identifying tumor-specific epigenetic alterations in primary rectal cancer specimens compared to matched normal mucosa from the same patient. Given the differences in the clinical treatment strategies between colon and rectal cancers and the higher degree of interindividual homogeneity between rectal cancers [13,14], we limited our analysis to primary rectal cancers. We specifically focused on the acetylation of histone 3 at lysine 27 (H3K27ac) due to its clear association with active gene transcription [15]. Genes with differential occupancy between tumor and mucosa were compared with gene expression data from an independent collection of rectal cancer, and selected genes were confirmed using immunohistochemistry.

## 2. Results

### 2.1. ChIP-Seq for H3K27ac from Primary Rectal Cancer and Adjacent Mucosa

**Figure 1:**
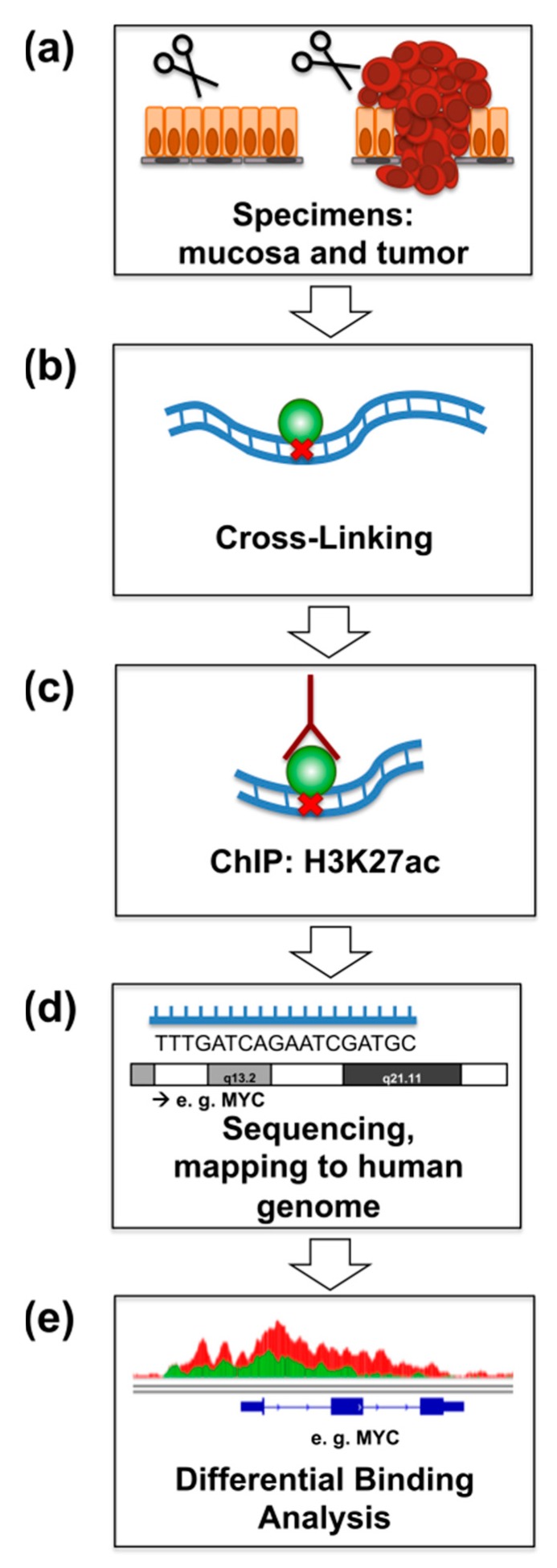
Schematic representation of the ChIP-seq experiments. (**a**) Specimens from four patients with rectal cancer and matched normal mucosa were obtained directly after surgical resection. (**b**) Samples were immediately incubated with formaldehyde for cross-linking. (**c**) ChIP was performed with an anti-H3K27ac-antibody. (**d**) After sequencing, fragments were mapped to the human reference genome (hg19). (**e**) Differential binding analysis identified differentially occupied regions near the transcriptional start site of genes.

**Figure 2:**
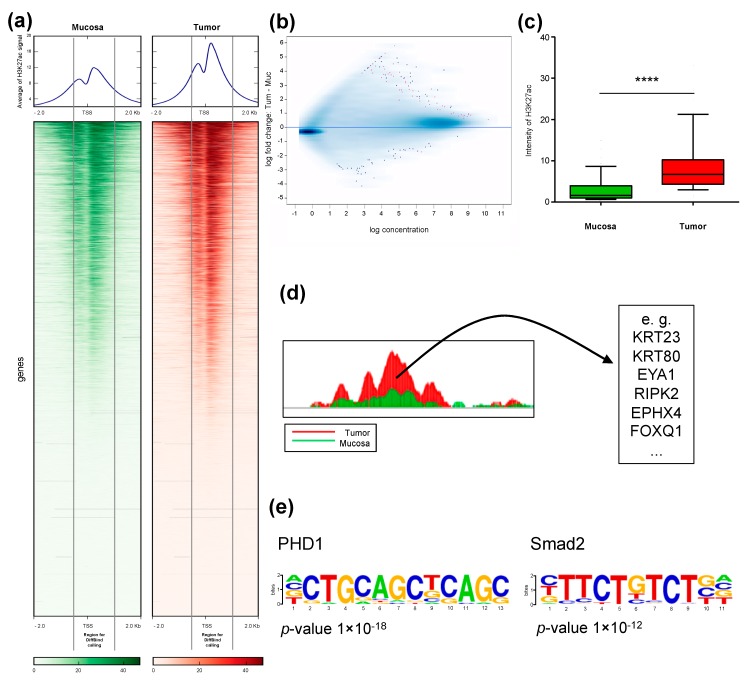
H3K27ac occupancy near the transcriptional start sites (TSS) is globally increased in rectal cancer compared to matched normal mucosa. (**a**) Heatmap and average of all peaks from patient P1 +/− 2 kb from TSS in descending order of mucosa and the same order for tumor. The marked area highlights the analyzed regions 500 bp upstream and 1 kb downstream from TSS. (**b**) Binding affinity plot: Tumor versus mucosa with a FDR <0.1. Genes with a significant difference are represented in pink. (**c**) Boxplot analysis of the 44 differentially bound regions in all four patients. Significance was calculated using the Mann–Whitney test. The whiskers represent the data from 10–90 percentiles. **** *p* < 0.0001. (**d**) Selected examples of the 44 genes with differential H3K27ac marking at the TSS. (**e**) Motifs discovered to be enriched in regions that gain H3K27ac in tumor samples.

We performed ChIP-seq of H3K27ac in rectal cancer and matched normal mucosa from four patients directly after surgical resection (Figure 1). Analysis of genome occupancy identified between 36,668 and 61,456 genomic regions that were enriched for H3K27ac in the tumors, and between 52,236 and 69,605 regions in mucosa samples. Although the overall number of peaks in the tumor samples was moderately decreased, a slight global increase in H3K27ac-intensity at transcriptional start sites (TSS) was observed in tumors compared to matched mucosa. A representative example for patient P1 is shown in the heatmap, where the overall pattern of occupancy was largely the same between the tumor and matched mucosa with a moderate increase directly adjacent to the TSS (Figure 2a). Importantly, the signal close to the TSS shows a typical pattern of enrichment with a characteristic gap directly at the transcriptional start site (TSS), which is referred to as the “nucleosome-free region” and is devoid of histone proteins due to the occupancy of RNA Polymerase II [16].

### 2.2. Identification of Differentially H3K27ac-Enriched TSS in Tumor and Mucosa

**Figure 3:**
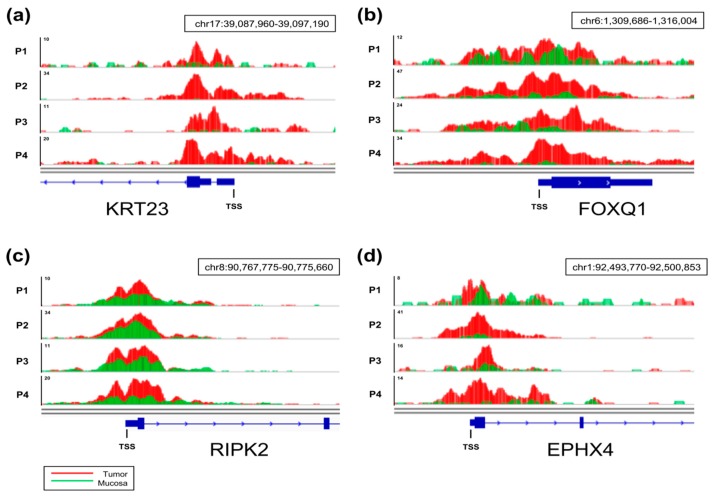
ChIP-seq tracks for H3K27ac occupancy. Representative tracks are displayed for *RIPK2* (**a**), *EPHX4* (**b**), *FOXQ1* (**c**), and *KRT23* (**d**) for all four patients (P1–P4). The corresponding gene is displayed in blue, and the transcriptional start site (TSS) is highlighted. The direction of transcription is marked with blue arrows within the gene. Tumors (red) and matched normal mucosa (green) are overlaid.

Given the general association of gene expression to H3K27ac occupancy near the TSS [17], we aimed to identify genes that displayed a specific increase in H3K27ac in tumors compared to mucosa by performing differential binding analysis (Figure 2b). By examining regions from 500 bp upstream to 1 kb downstream of annotated TSS, we identified 44 regions displaying significantly and differentially enriched H3K27ac occupancy in tumor samples compared to matched mucosa (Figure 2c). Examples of genes associated with the 44 differentially marked TSS are shown in Figure 2d, and a complete list is provided in Table S1. To further characterize the regions that were specifically gained in tumor samples, we performed motif enrichment analysis to unveil transcription factors that can preferentially bind to these regions. Interestingly, binding motifs for Hypoxia-inducible factor prolyl hydroxylase 1 (PHD1) and the TGFβ-responsive transcription factor SMAD2 were identified to be significantly enriched in tumor samples (Figure 2e). Examples of H3K27ac occupancy profiles at four of the identified genes are shown in Figure 3. Notably, increases in H3K27ac occupancy in tumor samples in comparison to adjacent mucosa are apparent in all four sample pairs.

### 2.3. Genes with Increased TSS-Proximal H3K27ac Are Differentially Expressed

**Figure 4:**
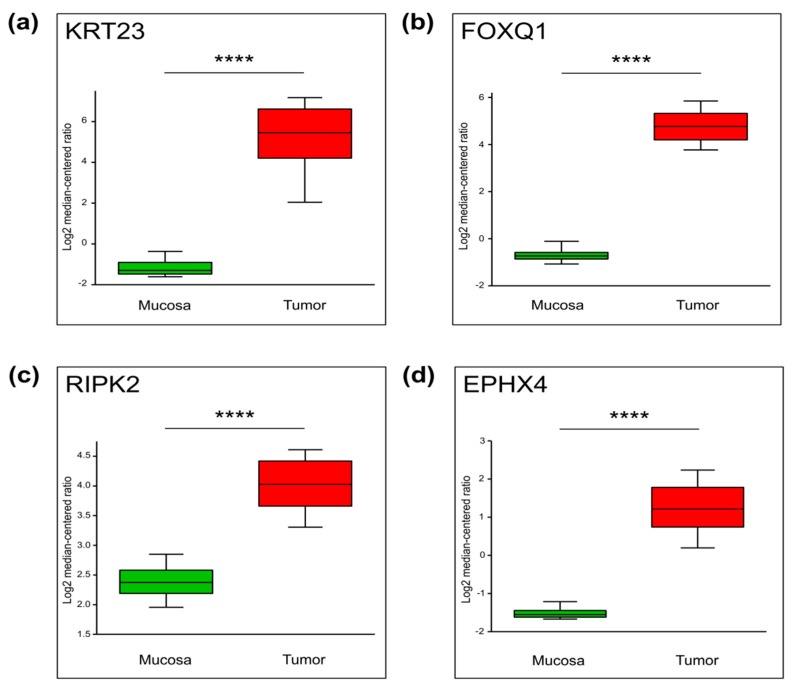
The mRNA levels of four selected differentially enriched genes which were obtained from publicly available datasets (Oncomine, Gaedcke Colorectal Statistics). Whiskers demonstrate the data from 10 to 90 percentiles. *N* = 130, **** *p* ≤ 0.0001. (**a**) *KRT23*, *p* = 9.06 × 10^−41^, (**b**) *FOXQ1*, *p* = 2.63 × 10^−63^, (**c**) *RIPK2*, *p* = 2.81 × 10^−42^, (**d**) *EPHX4*, *p* = 1.73 × 10^−16^.

We next hypothesized that genes with increased H3K27ac occupancy near the TSS may be frequently upregulated in rectal cancer. Therefore, we examined the expression of these identified genes in a publicly available dataset from a larger patient cohort. As shown in Figure 4, Table S1 and Figure S1, the mRNA levels of the vast majority of these genes are significantly increased in this independent patient cohort [18]. This confirms that the analysis of the active histone mark H3K27ac, even in a very small cohort of only four patients, is capable of providing valuable information about differential gene expression, which can be further validated in a larger, independent cohort of patients.

For further validation, four genes were selected: *RIPK2*, *FOXQ1*, *KRT23*, and *EPHX4*. These candidates were selected because they represent a selection of well-established proteins involved in oncological processes (i.e., RIPK2, KRT23, and FOXQ1) and putative novel targets (EPHX4), and because high-quality antibodies for immunohistochemistry were available.

### 2.4. Differential Cancer-Specific H3K27ac Occupancy Correlates with Tumor-Specific Changes in Protein Expression

**Figure 5:**
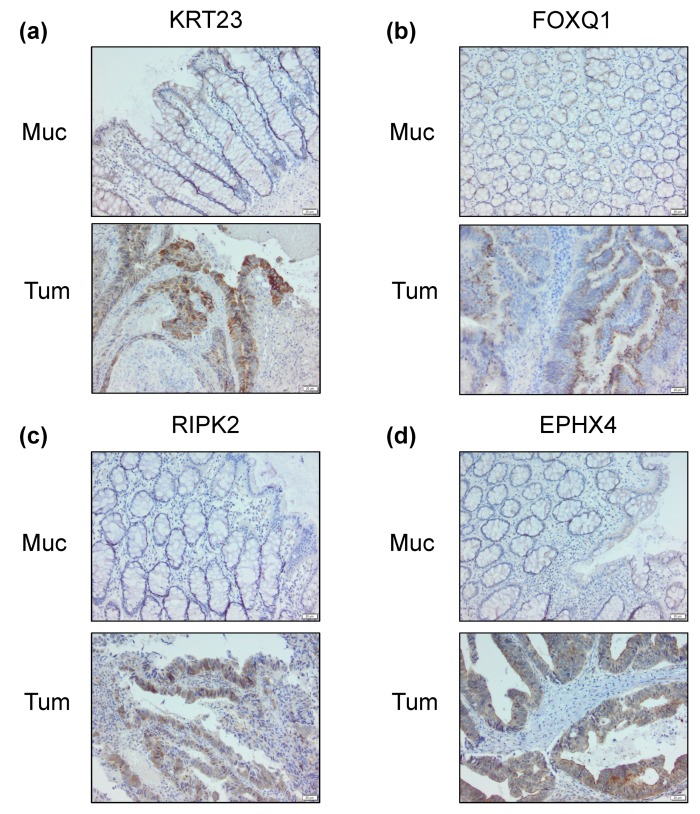
Immunohistochemical staining of the proteins encoded by the four selected genes with differential binding in both tumor and adjacent mucosa from the same patient. As an example, staining is shown for patient P4. KRT23 (**a**), FOXQ1 (**b**), RIPK2 (**c**) and EPHX4 (**d**). Scale bar: 20 μm.

Detailed analyses of gene expression data from bulk tumor samples have revealed that the differential expression of genes previously associated with patient prognosis are frequently identified due to the detection of expression in non-cancerous cells within the tumor stroma [19,20,21,22]. Thus, we sought to not only confirm the differential expression of *RIPK2*, *FOXQ1*, *KRT23* and *EPHX4* in the investigated tumor samples, but also examine whether the differential epigenetic marking of the genes was, indeed, specific for tumor cells and not from stromal contamination. Indeed, immunohistochemical analyses of archived formaldehyde-fixed paraffin-embedded tissue samples from the same patient samples examined via ChIP-seq not only confirmed that all four proteins are more highly expressed in the tumor samples relative to adjacent mucosa tissue, but that these proteins are also preferentially expressed in the epithelial compartment (Figure 5 and Figure S2).

### 2.5. H3K27ac Enrichment Marks Tumorigenic Gene Sets

**Figure 6:**
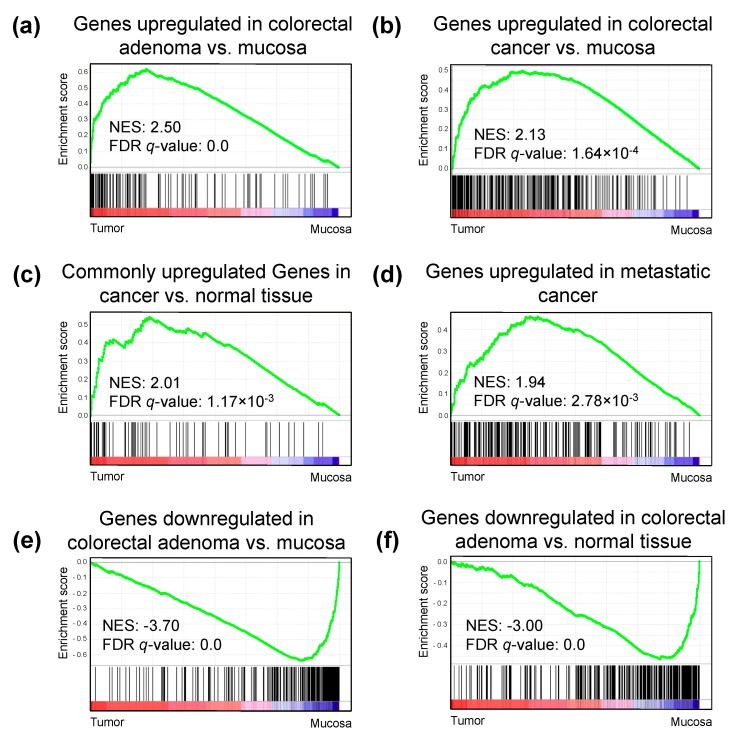
Gene set enrichment analysis (GSEA) plots depicting enriched pathways in tumor samples compared to normal mucosa. Reads per kilobase per million mapped reads (RPKM) values were used as expression values for each gene, and the analysis was performed for all C2 component datasets. Genes included in C2 pathways were ranked based on their enrichment in tumor versus mucosa (black lines). More genes significantly enriched in the tumor group results in a higher enrichment score (green line). (**a**–**d**) Top pathways that are enriched in the tumor group include signatures related to cancer, metastasis, and colorectal adenoma-specific signature. (**e**,**f**) Top pathways that are enriched in the mucosa group include pathways known to be downregulated in colorectal adenomas. NES, normalized enrichment score; FDR, false discovery rate.

To understand the underlying mechanisms and pathways associated with the differentially H3K27ac-marked regions, we performed gene set enrichment analysis (GSEA) using the TSS-proximal occupancy of H3K27ac as a proxy for gene expression. Interestingly, GSEA using the calculated intensities demonstrated an enrichment in gene sets correlated with colon and rectal adenoma and colorectal cancer compared to normal mucosa (Figure 6, Tables S2 and S3). Furthermore, genes generally associated with cancer development and metastasis were also upregulated in the tumor samples compared to mucosa. Moreover, gene sets enriched in the adjacent mucosa tissue compared to the tumor samples included genes associated with colorectal development and early cancer development. These findings further support the value of using H3K27ac-based ChIP-seq to impute gene expression in patient samples.

## 3. Discussion

Epigenome mapping data from chromatin modifications from primary cancer specimens is still rare due to several technical limitations. In particular, the ability to quickly process fresh surgical specimens for chromatin immunoprecipitation is impractical and requires a close interaction between the operating surgeon and laboratory researchers. Fanelli and colleagues sought to overcome these problems through the development of pathology tissue-chromatin immunoprecipitation (PAT-ChIP-seq) [23]. Using this approach, the authors compared the processing of fresh tissue with that of formalin-fixed paraffin-embedded material and demonstrated a high degree of concordance for H3K4me3 in the two conditions. The same group showed that a variation of this procedure, referred to as enhanced PAT-ChIP (EPAT-ChIP), which utilizes a limited reversal of crosslinking, was highly effective in the analysis of a normal colon sample as well as an archival breast cancer sample [24]. Similarly, Cejas and colleagues optimized the PAT-ChIP-seq protocol and successfully performed ChIP-seq for H3K4me2 in seven paraffin-embedded CRC patient samples. Subsequently, they compared the results to ChIP-seq data of six fresh frozen samples and also reported a high degree of concordance [25]. However, it is still unclear how well histone acetylation patterns remain constant following extended fixation and paraffin embedding.

While ChIP-seq has been performed and published in various primary cancer types [26,27,28,29,30,31], including just a few reports in colorectal cancer [25,32,33], to our knowledge, no ChIP-seq for H3K27ac in primary rectal cancer have been reported to date, and, importantly, not from tumor and matched normal mucosa of the same patients. Cohen and colleagues performed ChIP-seq of multiple CRC cell lines of different stages, two adenomas, four freshly isolated CRC and seven normal colon mucosa specimens [32]. The authors were able to show that enhancer regions marked by H3K27ac in CRC cell lines are correlated with cancer-specific gene expression. The comparison with primary CRC specimens showed a high correlation with regions identified in cell lines, both in ChIP-seq (genome occupancy) as well as in gene expression. Importantly, and consistent with our findings, one of the genes identified by Cohen and colleagues to be differentially marked in CRC compared to normal crypts was *FOXQ1*. Overexpression of *FOXQ1* was previously shown to increase tumor growth in a CRC xenograft mouse model [34].

The relevance of our findings is supported not only by the concordance with the findings of Cohen and colleagues that *FOXQ1* is upregulated in CRC, but also the fact that three of the four genes investigated for further confirmation, including *FOXQ1*, have been implicated in cancer development and progression. Like *FOXQ1*, *KRT23* has been connected with cancer progression in colorectal cancer [34,35]. In breast cancer, *RIPK2* was shown to promote breast cancer cell migration and invasion upstream of NFκB signaling [36]. Moreover, since *RIPK2* has been shown to play a role in inflammatory signaling in response to bacterial peptidoglycans [37], it is conceivable that it may also stimulate tumorigenic NFkB activity in sporadic colorectal cancer.

The biological relevance of our findings is further supported by the identification of upstream regulatory factors potentially controlling their expression. Notably, analysis of the promoter proximal regions of the differentially marked genes identified an enrichment in these sequences for binding of PHD1 and SMAD2. PHD1 is a modulator of HIF activity, whose role in cancer development is complex with somewhat contradictory results in colorectal cancer. On the one hand, overexpression of PHD1 was shown to decrease tumor growth in a murine xenograft model by reduction of HIF1α and VEGF levels [38]. On the other hand, inhibition of PHD1 sensitized HCT116 colorectal cancer cells to chemotherapy in a manner independent of HIF1α via direct interaction with p 53, [39]. The other identified transcription factor SMAD2 is a central component of the TGFβ signaling pathway and controls the expression of metastasis-associated genes [40].

## 4. Materials and Methods

### 4.1. Patients and Samples

We used cancer specimens and adjacent rectal mucosa from four patients with rectal cancer who underwent surgical resection in the Department of General, Visceral and Pediatric Surgery at the University Medical Center Goettingen. The study was conducted in accordance with the Helsinki Declaration and was approved by the ethics committee of the University Medical Center Goettingen (integrated in the KFO 179).

### 4.2. Chromatin Immunoprecipitation and Next Generation Sequencing

The biopsies from both tumor and adjacent normal rectum mucosa were taken immediately after surgical resection, diced into two- to three-millimeter pieces and immediately cross-linked. Chromatin immunoprecipitation (ChIP, Figure 1) was performed as previously described [41]. The dissociated tissue was cross-linked at room temperature for 15 min in 1% formaldehyde (prepared in PBS) and quenched with 125 mM glycine for 5 min. Samples were washed with PBS and stored at −150 °C. The cross-linked tissue was lysed and washed with nuclear preparation buffer (20 mM EDTA pH 8.0, 150 mM NaCl, 50 mM Tris-HCL pH 7.5, 0.5% NP-40 (*v*/*v*), 1% Triton-X-100 (*v*/*v*), 20 mM NaF, and protease inhibitor mix). We resuspended the nuclei in lysis buffer I (10 mM EDTA pH 8.0, 50 mM Tris-HCl pH 8.0, 1% SDS) and incubated for 15 min at 4 °C. Subsequently, equal volumes of lysis buffer II (150 mM NaCl, 20 mM EDTA pH 8.0, 50 mM Tris-HCL pH 8.0, 1% NP-40 (*v*/*v*), 20 mM NaF) were added to the nuclear pellet. The samples were sonicated with the Bioruptor^®^ Pico (Diagenode, Denville, NJ, USA) for 30 cycles (30 s on/off) and centrifuged at 12,000 g for 10 min. The chromatin extracts were precleared with 50% Sepharose 4B (GE Healthcare, Uppsala, Sweden, 17012001) in dilution buffer (150 mM NaCl, 20 mM EDTA pH 8.0, 50 mM Tris-HCl pH 8.0, 1% NP-40 (*v*/*v*), 20 mM NaF, 0.5% sodium deoxycholate (*w*/*v*) and protease inhibitors) and then immunoprecipitated overnight with 2 μg of either the anti-H3K27ac antibody (anti-H3K27ac, Diagenonde, C15910196) or non-specific IgG antibody (anti-IgG ab46540, Abcam, Cambridge, UK) as negative control. The next day, samples were precipitated with protein A-sepharose (GE17-0780-01, GE Healthcare) for 2 h at 4 °C. The immune complexes were washed with IP buffer (150 mM NaCl, 20 mM EDTA pH 8.0, 50 mM Tris-HCl pH 8.0, 1% NP-40 (*v*/*v*), 20 mM NaF, 0.5% sodium deoxycholate (*w*/*v*), SDS 0.1%), ChIP wash buffer (0.5 M LiCl, 20 mM EDTA pH 8.0, 0.1 mM Tris-HCl pH 8.5, 1% NP-40 (*v*/*v*), 1% sodium deoxycholate (*w*/*v*), 20 mM NaF), TE buffer (10 mM Tris-HCl pH 8.0, 1 mM EDTA pH 8.0) and treated with proteinase K overnight at 65 °C. The complexes were eluted with elution buffer (Tris 10 mM pH 8.0), and the DNA was extracted with phenol-chloroform. DNA was quantified with a Qubit^®^ dsDNA HS assay and a Qubit^®^ 2.0 Fluorimeter (Invitrogen, Life Technologies, CA, USA). Subsequently, 10 ng was used as input and 5 ng for H3K27ac of DNA for library preparation using MicroPlex Library Preparation Kit (Diagenode) and the preparation proceeded according to the manufacturer’s instructions. The size and quality control were performed with a Bioanalyzer 2100 (High Sensitivity DNA assay). Afterwards, single-end sequencing (51 bp) was run by the Transcriptome and Genome Analysis Laboratory using the HiSeq 2000 Illumina platform.

### 4.3. Statistical Analysis of ChIP-Seq Data

Quality control of the raw data was performed with FastQC (Galaxy version 0.69, Babraham bioinformatics, Cambridgeshire, UK). The FastQ files were mapped to the human reference genome (UCSC GRCh37/hg19) using Bowtie2 single-end very-sensitive mode [42]. Peak calling was performed using Model-based Analysis of ChIP-seq 2 (MACS2, Galaxy Version 2.1.1.20160309.0) for broad regions using respective inputs as background [43]. The minimum FDR cut-off value for peak detection was defined as *q* < 0.05 and duplicates were removed. The visualization of the ChIP-seq data was done with Integrative Genomics Viewer (IGV) [44]. Heatmaps centered around the TSS were generated with DEEPTOOLS/2.4.0 and display 2000 bp up- and downstream in descending order of signal intensity [45].

We performed differential binding analysis using the Bioconductor R package Diffbind run on R version 3.3.1 to identify regions with enhanced H3K27ac binding −500 bp and +1000 bp up- and downstream of all TSS [28]. All four patient samples were treated as replicates. The associated genes with the H3K27ac-enriched regions from DiffBind were identified using the Genomic Regions Enrichment of Annotations Tool (GREAT) [46] with the setting single nearest gene. DEEPTOOLS/2.4.0 computeMatrix was used to quantify the density of H3K27ac 500 bp upstream of the TSS and 1000 bp downstream. Motif analysis was performed using HOMER/4.8 against shuffled sequences as background [47]. Boxplots were plotted using Graph Pad Prism 5 and significance was calculated using the Mann–Whitney test.

Gene set enrichment analysis was performed using the mean reads per kilobase per million mapped reads (RPKM) values of H3K27ac calculated at the TSS of the respective gene using default settings (1000 permutations) and taking patient samples as replicates [48].

### 4.4. Immunohistochemistry

Immunohistochemistry was performed with paraffin-embedded tissues cut into 2 μm sections. Tissue slides were deparaffinated by adding two times xylol for 10 min and washed with decreasing alcohol concentrations. A heat-induced epitope retrieval was performed at 100 °C and pH 8.5 in Tris-EDTA for 45 min. Afterwards, the samples were incubated with 3% H_2_O_2_ for 15 min followed by 5% BSA for 10 min. The antibodies were added to the tissue at room temperature as follows: anti-FOXQ1 (1:100 diluted, two hour incubation, Abcam, ab51340), anti-KRT23 (1:200 dilution, one hour incubation, Sigma Aldrich; Taufkirchen, Germany, HPA016959), anti-RIPK2 (1:50 dilution, two hour incubation, Sigma Aldrich; HPA015273) and anti-EPHX2 (1:50 dilution, one hour incubation, Sigma Aldrich; HPA035067). Visualization of the enzymatic reactivity was operated with the secondary antibody EnVision (concentrated, HRP-coupled, Dako, Hamburg, Germany) incubated for 35 min, Chromogen (DAB 1:25 diluted, ImmunnoLogic, Amsterdam, The Netherlands) for eight min and hemalaun (Thermo Scientific, Waltham, MA, USA) for two min at room temperature. The tissues were washed for five min and treated with increasing alcohol concentrations. Xylol was added for two min and the preservation performed with Vitroclud.

## 5. Conclusions

Thus, while further analyses will be necessary to determine the relationship between the different target genes identified and their upstream regulatory pathways in tumorigenesis, our findings demonstrate the utility of performing genome-wide occupancy studies for active chromatin marks in primary rectal cancer and matched normal mucosa in order to identify meaningful, tumor-specific molecular epigenetic alterations. Importantly, we were the first to demonstrate that the observed increased occupancy of H3K27ac at tumor-specific proximal promoter regions is associated with increased gene expression at both the mRNA and protein levels not only in primary tissue, but also in an independent dataset. Further analyses will be necessary to identify experimental approaches which will allow the utilization of such genome-wide technologies on a more routine basis to uncover prognostic or diagnostic tumor-specific signatures.

## Supplementary Materials

The following are available online at https://www.mdpi.com/2072-6694/11/8/1142/s1, Figure S1: Graphical presentation of the mRNA levels of 12 additional differentially enriched genes (from Oncomine, Gaedcke Colorectal Statistics), Figure S2: Immunohistochemical staining of KRT23 (a), FOXQ1 (b), RIPK2 (c), and EPHX4 for patients P1–P3, Table S1: List of the 44 genes with differential H3K27ac occupancy at TSS, Table S2: Table of GSEA top 50 gene sets enriched in phenotype tumor compared to mucosa, Table S3: Table of GSEA top 50 gene sets enriched in phenotype mucosa compared to tumor.
