# Supplementary material for: Epigenome Mapping Identifies Tumor-Specific Gene Expression in Primary Rectal Cancer
Source: Cancers (Basel). 2019 Aug 9;11(8):1142. doi: 10.3390/cancers11081142 (PMC6721540; doi:10.3390/cancers11081142)
Supplement: Supplementary file 1 [file cancers-11-01142-s001.pdf]

Supplementary Material: Epigenome Mapping Identifies Tumor-Specific Gene Expression in Primary Rectal Cancer

Hannah Flebbe, Feda H. Hamdan, Vijayalakshmi Kari, Julia Kitz, Jochen Gaedcke, B. Michael Ghadimi, Steven A. Johnsen and Marian Grade

Table S1. List of the 44 genes with differential H3K27ac occupancy at TSS. Also shown are the *p*-values of the corresponding gene expression differences between tumor and mucosa samples from Gaedcke Colorectal Statistics (Oncomine). n.a., not available.

Number	Name	*p*-Value for Gene Expression Differences
1	CASC21	n. a.
2	KRT23	9.06×10^{-41}
3	MRPL23	3.80×10^{-10}
4	PLA2G2E	0.043
5	F7	3.16×10^{-10}
6	LINCO1219	n. a.
7	BMP7	7.14×10^{-16}
8	PRSS23	2.17×10^{-17}
9	RETN	1.36×10^{-18}
10	TREM1	2.75×10^{-21}
11	NR_125862	n. a.
12	EPHX4	1.73×10^{-16}
13	MMP9	n. a.
14	LINCO1101	n. a.
15	RBP2	5.92×10^{-4}
16	CXCL6	5.73×10^{-17}
17	LINCO1615	n. a.
18	HAL	6.86×10^{-9}
19	MCEMP1	2.87×10^{-22}
20	PRAP1	4.91×10^{-25}
21	EYA1	3.44×10^{-4}
22	CHI3L1	3.42×10^{-40}
23	LGR5	1.32×10^{-10}
24	ADGRG3	7.05×10^{-13}
25	ETV4	2.898×10^{-44}
26	KRT80	1.18×10^{-46}
27	CTHRC1	3.71×10^{-30}
28	TNFRSF10C	1.10×10^{-19}
29	PILRB	0.785
30	FOXQ1	2.63×10^{-63}
31	LOC254896	n. a.
32	C6orf223	6.49×10^{-24}
33	MROH6	7.55×10^{-18}
34	ASCL2	8.24×10^{-52}
35	EVA1A	3.65×10^{-26}
36	ARNTL2	1.68×10^{-35}
37	LY6E	6.16×10^{-28}
38	SERPINA1	0.993
39	SNTB1	2.32×10^{-33}
40	TGFBI	1.28×10^{-50}
41	PVT1	7.75×10^{-29}
42	S100P	5.81×10^{-7}
43	CXCL1	6.45×10^{-31}
44	RIPK2	2.81×10^{-42}

Table S2. Table of GSEA top 50 gene sets enriched in phenotype tumor compared to mucosa. NES, normalized enrichment score; FDR, false discovery rate.

NO.	Name	Size	NES	FDR *q*-Value
1	NIKOLSKY_BREAST_CANCER_8Q12_Q22_AMPLICON	105	2.67	0.000
2	NIKOLSKY_BREAST_CANCER_8Q23_Q24_AMPLICON	135	2.57	0.000
3	SABATES_COLORECTAL_ADENOMA_UP	117	2.50	0.000
4	NIKOLSKY_BREAST_CANCER_20Q12_Q13_AMPLICON	117	2.49	0.000
5	RHODES_UNDIFFERENTIATED_CANCER	67	2.19	0.000
6	GRADE_COLON_AND_RECTAL_CANCER_UP	269	2.13	0.000
7	VECCHI_GASTRIC_CANCER_EARLY_UP	390	2.11	0.000
8	BOYAULT_LIVER_CANCER_SUBCLASS_G3_UP	181	2.07	0.000
9	ROSTY_CERVICAL_CANCER_PROLIFERATION_CLUSTER	134	2.06	0.000
10	CHEMNITZ_RESPONSE_TO_PROSTAGLANDIN_E2_UP	134	2.05	0.000
11	RHODES_CANCER_META_SIGNATURE	63	2.01	0.001
12	REACTOME_INFLUENZA_LIFE_CYCLE	131	2.00	0.001
13	REACTOME_PROCESSING_OF_CAPPED_INTRON_CONTAINING_PRE_MRNA	132	1.99	0.002
14	REACTOME_AMYLOIDS	68	1.98	0.002
15	REACTOME_3_UTR_MEDIATED_TRANSLATIONAL_REGULATION	103	1.97	0.002
16	SOTIRIOU_BREAST_CANCER_GRADE_1_VS_3_UP	143	1.97	0.002
17	REACTOME_MEIOTIC_RECOMBINATION	77	1.97	0.002
18	FISCHER_DREAM_TARGETS	902	1.95	0.003
19	PEART_HDAC_PROLIFERATION_CLUSTER_DN	73	1.94	0.003
20	KEGG_RIBOSOME	85	1.94	0.003
21	BURTON_ADIPOGENESIS_PEAK_AT_24HR	40	1.94	0.003
22	BIDUS_METASTASIS_UP	202	1.94	0.003
23	CAIRO_HEPATOBLASTOMA_CLASSES_UP	567	1.93	0.003
24	REACTOME_RNA_POL_I_TRANSCRIPTION	82	1.93	0.003
25	REACTOME_PEPTIDE_CHAIN_ELONGATION	83	1.93	0.003
26	REACTOME_MRNA_PROCESSING	152	1.92	0.003
27	REACTOME_RNA_POL_I_PROMOTER_OPENING	57	1.92	0.003
28	WU_APOPTOSIS_BY_CDKN1A_VIA_TP53	52	1.92	0.003
29	REACTOME_SRP_DEPENDENT_COTRANSLATIONAL_PROTEIN_TARGETING_TO_MEMBRANE	104	1.92	0.003
30	LINDSTEDT_DENDRITIC_CELL_MATURATION_B	53	1.91	0.003
31	REACTOME_TRANSLATION	141	1.91	0.003
32	DUNNE_TARGETS_OF_AML1_MTG8_FUSION_UP	47	1.90	0.003
33	KANG_DOXORUBICIN_RESISTANCE_UP	50	1.90	0.003
34	BENPORATH_PROLIFERATION	134	1.90	0.004
35	REACTOME_INFLUENZA_VIRAL_RNA_TRANSCRIPTION_AND_REPLICATION	98	1.90	0.004
36	ASTIER_INTEGRIN_SIGNALING	51	1.88	0.005
37	LI_AMPLIFIED_IN_LUNG_CANCER	159	1.87	0.005
38	WINNEPENNINCKX_MELANOMA_METASTASIS_UP	150	1.87	0.005
39	CROMER_TUMORIGENESIS_UP	51	1.87	0.005
40	REACTOME_MRNA_SPLICING	104	1.87	0.005
41	REACTOME_DEPOSITION_OF_NEW_CENPA_CONTAINING_NUCLEOSOMES_AT_THE_CENTROMERE	58	1.87	0.005
42	REACTOME_FORMATION_OF_THE_TERNARY_COMPLEX_AND_SUBSEQUENTLY_THE_43S_COMPLEX	48	1.87	0.005
43	SARRIO_EPITHELIAL_MESENCHYMAL_TRANSITION_UP	167	1.87	0.006
44	PUJANA_BRCA2_PCC_NETWORK	399	1.86	0.005
45	TIEN_INTESTINE_PROBIOTICS_6HR_UP	55	1.85	0.007
46	PRAMOONJAGO_SOX4_TARGETS_UP	50	1.84	0.007
47	VANTVEER_BREAST_CANCER_METASTASIS_DN	110	1.84	0.007
48	KOBAYASHI_EGFR_SIGNALING_24HR_DN	241	1.84	0.007
49	DANG_MYC_TARGETS_UP	137	1.84	0.008
50	ISHIDA_E2F_TARGETS	50	1.83	0.008

Table S3. Table of GSEA top 50 gene sets enriched in phenotype mucosa compared to tumor. NES, normalized enrichment score; FDR, false discovery rate.

NO.	Name	Size	NES	FDR q-value
1	SABATES_COLORECTAL_ADENOMA_DN	230	−3.70	0.000
2	VECCHI_GASTRIC_CANCER_EARLY_DN	279	−3.00	0.000
3	SMID_BREAST_CANCER_LUMINAL_A_UP	69	−2.69	0.000
4	SMID_BREAST_CANCER_RELAPSE_IN_LUNG_DN	31	−2.32	0.000
5	NAKAYAMA_SOFT_TISSUE_TUMORS_PCA2_DN	69	−2.21	0.001
6	DELYS_THYROID_CANCER_DN	210	−2.20	0.001
7	BOQUEST_STEM_CELL_UP	245	−2.13	0.001
8	RICKMAN_HEAD_AND_NECK_CANCER_F	36	−1.92	0.011
9	WONG_ENDMETRIUM_CANCER_DN	72	−1.88	0.016
10	REACTOME_NCAM1_INTERACTIONS	32	−1.85	0.019
11	CAIRO_HEPATOBLASTOMA_CLASSES_DN	185	−1.78	0.032
12	BROWNE_HCMV_INFECTION_2HR_UP	36	−1.78	0.030
13	TURASHVILI_BREAST_DUCTAL_CARCINOMA_VS_LOBULAR_NORMAL_DN	60	−1.78	0.029
14	WINNEPENNINCKX_MELANOMA_METASTASIS_DN	38	−1.76	0.034
15	TOMLINS_PROSTATE_CANCER_DN	40	−1.74	0.037
16	SIMBULAN_PARP1_TARGETS_UP	31	−1.73	0.040
17	BERTUCCI_INVASIVE_CARCINOMA_DUCTAL_VS_LOBULAR_DN	39	−1.72	0.040
18	ISSAEVA_MLL2_TARGETS	54	−1.63	0.076
19	KEGG_RETINOL_METABOLISM	35	−1.63	0.076
20	KEGG_DRUG_METABOLISM_CYTOCHROME_P450	45	−1.63	0.073
21	KEGG_NEUROACTIVE_LIGAND_RECEPTOR_INTERACTION	156	−1.63	0.070
22	CHEBOTAEV_GR_TARGETS_UP	72	−1.62	0.070
23	NABA_CORE_MATRISOME	210	−1.62	0.068
24	VANHARANTA_UTERINE_FIBROID_DN	63	−1.62	0.067
25	MIKKELSEN_MCV6_HCP_WITH_H3K27ME3	298	−1.60	0.072
26	KEGG_ALDOSTERONE_REGULATED_SODIUM_REABSORPTION	38	−1.59	0.080
27	NAKAJIMA_MAST_CELL	39	−1.58	0.081
28	REN_ALVEOLAR_RHABDOMYOSARCOMA_UP	81	−1.58	0.082
29	SWEET_LUNG_CANCER_KRAS_DN	374	−1.57	0.085
30	REACTOME_GABA_RECEPTOR_ACTIVATION	38	−1.56	0.091
31	EBAUER_MYOGENIC_TARGETS_OF_PAX3_FOXO1_FUSION	38	−1.56	0.089
32	KONDO_PROSTATE_CANCER_WITH_H3K27ME3	78	−1.56	0.087
33	KEGG_ECM_RECEPTOR_INTERACTION	73	−1.53	0.099
34	SERVITJA_ISLET_HNF1A_TARGETS_UP	138	−1.53	0.101
35	BENPORATH_PRC2_TARGETS	468	−1.51	0.112
36	NABA_ECM_GLYCOPROTEINS	154	−1.50	0.116
37	REACTOME_NUCLEAR_RECEPTOR_TRANSCRIPTION_PATHWAY	42	−1.49	0.121
38	LEIN_NEURON_MARKERS	52	−1.48	0.129
39	GERHOLD_ADIPOGENESIS_UP	46	−1.48	0.133
40	HOWLIN_CITED1_TARGETS_1_DN	35	−1.47	0.137
41	REACTOME_PHOSPHOLIPASE_C_MEDIATED_CASCADE	44	−1.47	0.136
42	ACEVEDO_FGFR1_TARGETS_IN_PROSTATE_CANCER_MODEL_DN	258	−1.46	0.138
43	LIU_PROSTATE_CANCER_DN	434	−1.42	0.181
44	BOYAULT_LIVER_CANCER_SUBCLASS_G123_DN	46	−1.42	0.181
45	REACTOME_MUSCLE_CONTRACTION	35	−1.41	0.189
46	NABA_COLLAGENS	35	−1.39	0.213
47	KEGG_METABOLISM_OF_XENOBIOTICS_BY_CYTOCHROME_P450	45	−1.39	0.209
48	NABA_ECM_AFFILIATED	123	−1.37	0.232
49	HOWLIN_PUBERTAL_MAMMARY_GLAND	59	−1.37	0.230
50	YAO_HOXA10_TARGETS_VIA_PROGESTERONE_UP	68	−1.37	0.227

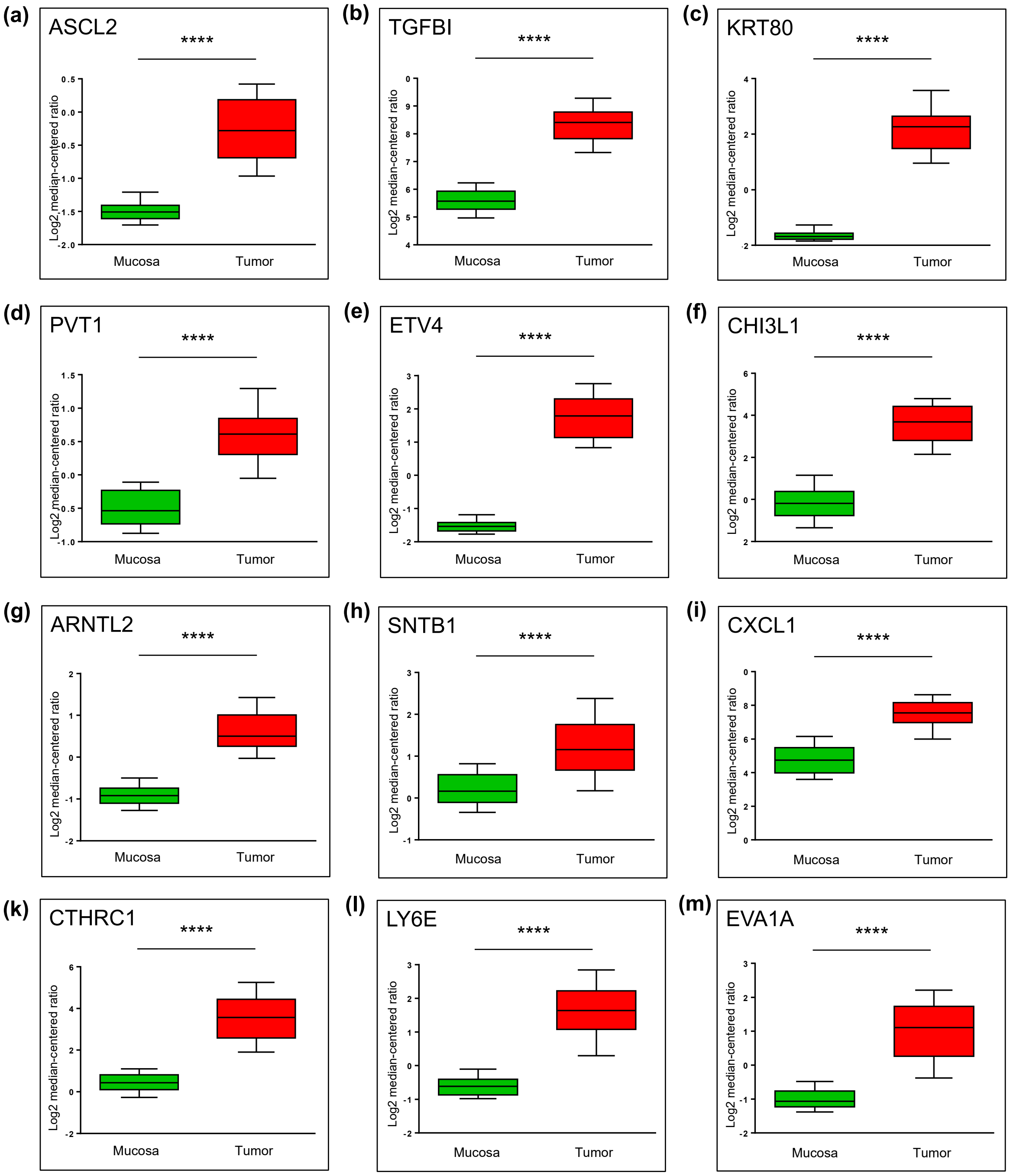

Figure S1. Graphical presentation of the mRNA levels of 12 additional differentially enriched genes (from Oncomine, Gaedcke Colorectal Statistics). Whiskers demonstrate the data from 10 to 90 percentiles. $n = 130$. p-values are listed in Table S1. **** $p < 0.0001$.

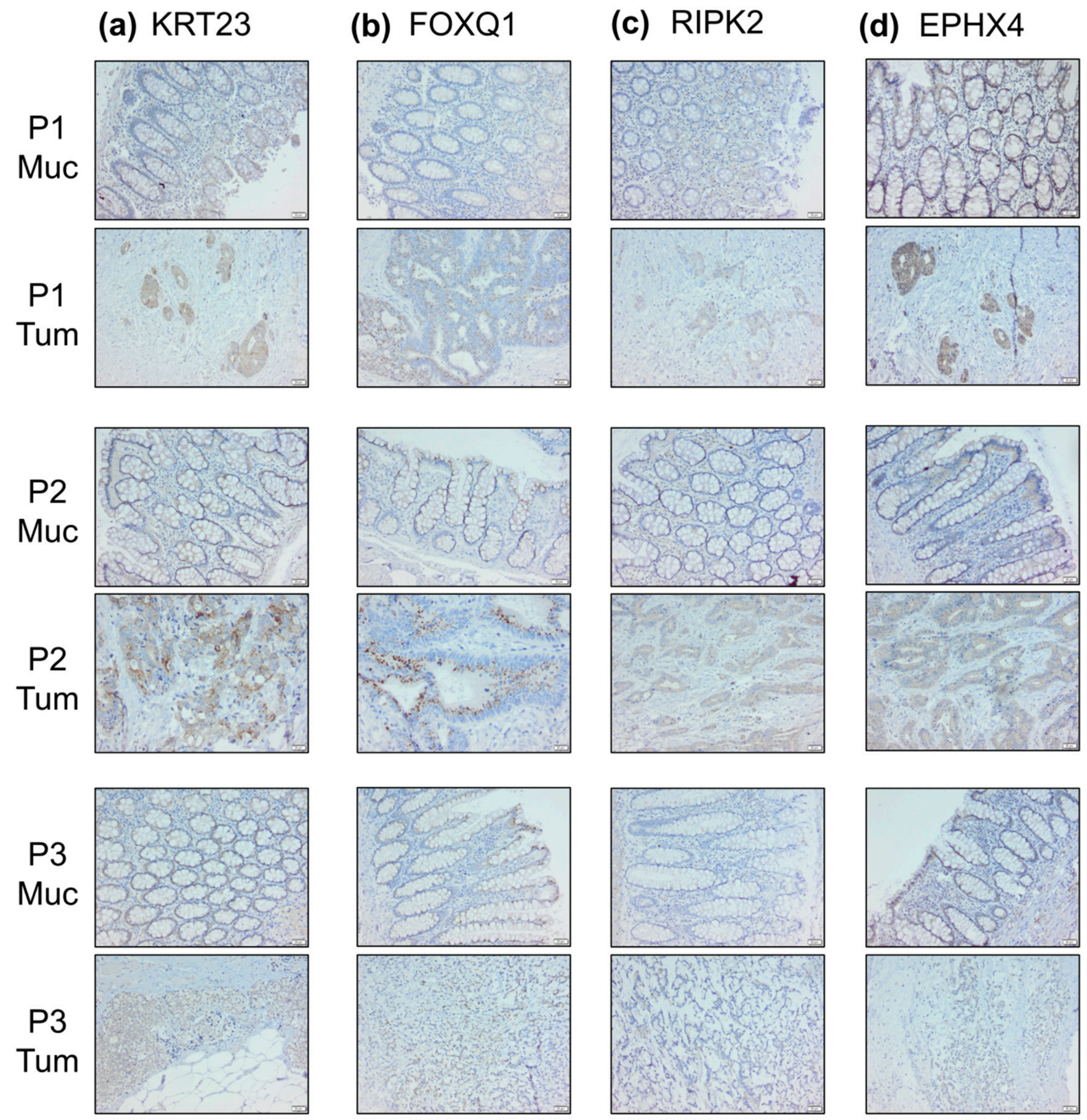

Figure S2. Immunohistochemical staining of KRT23 (**a**), FOXQ1 (**b**), RIPK2 (**c**), and EPHX4 (**d**) for patients P1–P3. Scale bar: 20 µm.

© 2019 by the authors. Licensee MDPI, Basel, Switzerland. This article is an open access article distributed under the terms and conditions of the Creative Commons Attribution (CC BY) license (http://creativecommons.org/licenses/by/4.0/).
